# Identification of cachexia in lung cancer patients with an ensemble learning approach

**DOI:** 10.3389/fnut.2024.1380949

**Published:** 2024-05-30

**Authors:** Pingping Jia, Qianqian Zhao, Xiaoxiao Wu, Fangqi Shen, Kai Sun, Xiaolin Wang

**Affiliations:** Department of Clinical Nutrition, Beijing Shijitan Hospital, Capital Medical University, Beijing, China

**Keywords:** lung cancer, cachexia, weight loss, ensemble learning, cohort study

## Abstract

**Objective:**

Nutritional intervention prior to the occurrence of cachexia will significantly improve the survival rate of lung cancer patients. This study aimed to establish an ensemble learning model based on anthropometry and blood indicators without information on body weight loss to identify the risk factors of cachexia for early administration of nutritional support and for preventing the occurrence of cachexia in lung cancer patients.

**Methods:**

This multicenter study included 4,712 lung cancer patients. The least absolute shrinkage and selection operator (LASSO) method was used to obtain the key indexes. The characteristics excluded weight loss information, and the study data were randomly divided into a training set (70%) and a test set (30%). The training set was used to select the optimal model among 18 models and verify the model performance. A total of 18 machine learning models were evaluated to predict the occurrence of cachexia, and their performance was determined using area under the curve (AUC), accuracy, precision, recall, F1 score, and Matthews correlation coefficient (MCC).

**Results:**

Among 4,712 patients, 1,392 (29.5%) patients were diagnosed with cachexia based on the framework of Fearon et al. A 17-variable gradient boosting classifier (GBC) model including body mass index (BMI), feeding situation, tumor stage, neutrophil-to-lymphocyte ratio (NLR), and some gastrointestinal symptoms was selected among the 18 machine learning models. The GBC model showed good performance in predicting cachexia in the training set (AUC = 0.854, accuracy = 0.819, precision = 0.771, recall = 0.574, F1 score = 0.658, MCC = 0.549, and kappa = 0.538). The abovementioned indicator values were also confirmed in the test set (AUC = 0.859, accuracy = 0.818, precision = 0.801, recall = 0.550, F1 score = 0.652, and MCC = 0.552, and kappa = 0.535). The learning curve, decision boundary, precision recall (PR) curve, the receiver operating curve (ROC), the classification report, and the confusion matrix in the test sets demonstrated good performance. The feature importance diagram showed the contribution of each feature to the model.

**Conclusions:**

The GBC model established in this study could facilitate the identification of cancer cachexia in lung cancer patients without weight loss information, which would guide early implementation of nutritional interventions to decrease the occurrence of cachexia and improve the overall survival (OS).

## 1 Introduction

Lung cancer, the most prevalent malignancy globally and a primary contributor to cancer-related fatalities (constituting ~18% of all cancer deaths) (1), was often accompanied by cachexia. Cachexia is a multifactorial and multi-organ syndrome prevalent in the late stages of chronic conditions (2), especially among cancer patients. Cachexia, a metabolic syndrome, is characterized by loss of muscle mass, with or without concurrent loss of fat mass, and diminished bodily functions. This condition is frequently attributed to an inflammatory state or reduced food intake (2). It manifests in nearly 50% of lung cancer patients and indirectly contributes to at least 20% deaths of all cancer patients (3, 4). Cachexia during cancer treatment poses a severe complication as it is linked to increased chemotherapy-related side effects, fewer completed cycles of chemotherapy, and reduced survival rates (5, 6).

Despite the widespread occurrence of cachexia in clinical practice, addressing its prevention, early identification, and intervention remains a challenge. The impact of cancer cachexia on quality of life, treatment-related toxicity (7, 8), physical function impairment, and mortality was well documented. However, focusing on weight loss to establish a clinically meaningful definition for the diagnosis of cachexia had proven challenging because the failure of patients in recalling their weight history can lead to lack of accurate weight loss information. Early identification and management of cachexia is critical for its prevention. Moreover, cachexia is characterized by not just weight loss but also loss of body composition and body function (9). Importantly, emaciation is a late symptom of cachexia and a characteristic of its advanced phase. Although attempts to more comprehensively define cachexia through body composition, body function, and molecular biomarkers were promising, it had not been routinely incorporated into clinical practice (9–11). To date, there are no effective pharmacological interventions that can completely reverse cachexia. Adequate and early nutritional support remains the mainstay of cachexia treatment (12).

In the established diagnostic framework (12), cachexia is defined as an involuntary weight loss of more than 5% or a body mass index (BMI) of <20 kg/m^2^, with sustained weight loss of more than 2% within the past 6 months. Additionally, it included cases of sarcopenia combined with sustained weight loss of more than 2%. Cancer cachexia is categorized into three stages: pre-cachexia, cachexia, and refractory cachexia. Notably, refractory cachexia has been the focus in numerous clinical trials for novel interventional drugs (12). However, demonstrating its therapeutic efficacy in this stage is challenging due to the catabolic state's resistance to anticancer therapy, the low performance state, and a prognosis of survival of <3 months. Currently, the focus of academic research had shifted from advanced cachexia to the etiology of cachexia (13). Evidence has shown that different drivers of inflammation, metabolism, and neuro-modulatory can initiate processes that eventually led to advanced cachexia (14).

This study aimed to use the nutrition-related parameters without weight loss information to establish an ensemble learning system for predicting and diagnosing the occurrence of cachexia in lung cancer patients before weight loss or a lack of weight loss information, which will be beneficial for administrating early nutritional support and improving the overall survival (OS) of lung cancer patients.

## 2 Methods

### 2.1 Population

All patients were enrolled from the Investigation on Nutrition Status and its Clinical Outcome of Common Cancers (INSCOC) project, which enrolled participants from various clinical centers across China, starting in 2013. The trial registration can be accessed at the Chinese Clinical Trial Registry under the identifier: ChiCTR1800020329. The design, methods, and development of the INSCOC study were conducted as previously described (15).

A total of 5,160 patients diagnosed with lung cancer through pathological examination were admitted for cancer treatment starting in 2013. After excluding 188 participants due to the presence of other primary tumors and 260 participants who were unable to recall weight loss information, we finally included 4,712 patients. Prior to the initiation of the study, all selected patients signed informed consent forms within 48 h of hospital admission. The study protocol adhered to the ethical guidelines outlined in the 1975 Declaration of Helsinki and received approval from the Institutional Review Committee of Beijing Shijitan Hospital.

### 2.2 Baseline data collection

The baseline information of the patients encompassed age, sex, smoking history, comorbidities such as diabetes, hypertension, and anemia, a family history of cancer, and weight loss (with at least 1 month of weight loss information recall). In addition, hematological examination indicators such as creatinine, total protein, albumin, prealbumin, blood urea nitrogen (BuN), total bilirubin (TBil), direct bilirubin (DBil), aspartate aminotransferase (AST), alanine aminotransferase (ALT), blood glucose, hemoglobin, red blood cell (RBC), white blood cell (WBC), neutrophils, lymphocytes, and platelets (PLT) were also included in the study. A comprehensive interview, conducted by a dietitian or clinician, was carried out with each patient to collect information on recent nutrition. This included assessments such as the Nutritional Risk Screening 2002 (NRS-2002), the Karnofsky Performance Score (KPS), and the European Organization for Research and Treatment of Cancer QLQ-C30 score (QLQ-C30). The NRS-2002 served as a tool for nutritional risk screening, established through the analysis of 128 trials. It had been endorsed by the European Society for Clinical Nutrition and Metabolism (ESPEN) for utilization in clinical settings (16). NRS2002-partial, defined as NRS2002 removing weight loss information, was included in the model. In addition, we determined the value of appendicular skeletal muscle mass (ASM) based on earlier research: 0.193^*^body weight (kg) + 0.107^*^height (cm) – 4.157^*^sex (male: 1, female: 2) – 0.037 (years) – 2.631. The appendicular skeletal muscle index (ASMI) was defined as ASM (kg)/height^2^ (m^2^). Patients were classified as having low muscle mass when ASMI was <7 for men or 5.4 for women (17, 18). According to the etiologic criteria of the Global Leadership Initiative on Malnutrition (GLIM) (19), we included gastrointestinal symptoms, reduced food intake, and inflammatory status. Gastrointestinal symptoms include anorexia, nausea, and vomiting. Four inflammatory indicators, namely, neutrophil-to-lymphocyte ratio (NLR), advanced lung cancer inflammation index (ALI), platelet-to-lymphocyte ratio (PLR), and nutritional risk index (NRI), were included. The ALI was calculated from the following formula: BMI^*^Albumin/NLR. The NRI is calculated from the following formula: 1.519^*^Albumin + 41.7^*^present body weight/ideal body weight. The ideal body weight is defined as (height – 100)^*^0.9. Anthropometric measurements have been widely used as nutritional indicators to evaluate the nutritional status of individuals and populations (20). This study also included anthropometric indicators: height, weight, BMI (weight/height^2^), mid-arm circumference (MAC); triceps skinfold thickness (TSF); hand grip strength (HGS); mid-arm muscle circumference (MAMC); and calf circumference (CC). Mid-arm circumference (MAC) was measured to the nearest 0.5 cm midway between the acromion and the olecranon. Triceps skin fold (TSF) was determined with the skin fold thickness meter. Mid-arm muscle circumference (MAMC) was calculated by using the following formula: MAMC (mm) = MAC (mm) – [3.14 × TSF (mm)]. Hand grip strength (HGS) was measured by using a hand dynamometer, and calf circumference (CC) was assessed at the thickest part of the calf with a flexible anthropometric tape.

### 2.3 Statistical analysis

Data of continuous variable were expressed as median (*M*) and inter-quartile range (IQR), while data of categorical variables were presented as frequency and percentage. Group comparisons for patients' tumor characteristics used the chi-squared test for categorical variables and independent-samples *t*-test for continuous variables. The analyses were performed using Python Version 3.7.3 and IBM SPSS (Version 26.0; IBM, Armonk, NY, USA). Statistical significance was defined as a *P*-value of <0.05 (two-sided).

## 3 Results

### 3.1 Baseline characteristics

**Figure 1:**
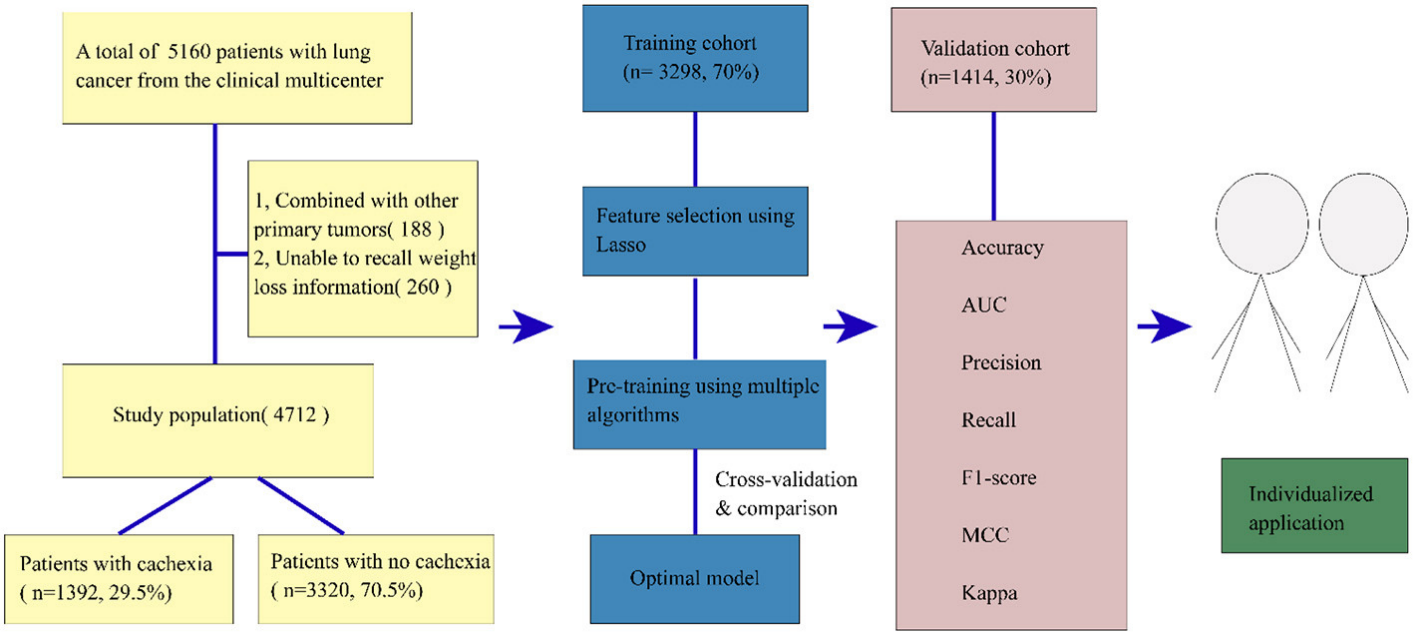
The study flowchart. AUC, area under the curve; MCC, Matthews correlation coefficient.

A total of 4,712 lung cancer patients from a multicenter study were analyzed. A flow chart of participant selection is shown in Figure 1 and the baseline characteristics of the study population are displayed in Table 1, encompassing routinely available data on demographics, pathology, tumor stages, blood indices, anthropometric parameters, weight loss, feeding situation, gastrointestinal symptoms, and the physical performance status of patients. A total of 1,392 patients were found to have cachexia, accounting for 29.5% of all patients, which was consistent with previous data (21). In addition, in our study data, the prevalence of cachexia in advanced patients was 49.7, which was consistent with data of previous studies (50%) (3). There were 2,410 (51.1%) patients with non-small cell lung cancer (NSCLC), 602 (12.8%) with small cell lung cancer (SCLC), and 1,700 (36.1%) patients with lung cancer who lacked a clear pathological classification. In our study, the mean age of patients was 61 [54, 67] years, with a higher number of male patients (66.6%, *n* = 3,139). Among the patients, 59.8% were smokers, and a substantial number (49.7%) exhibited distant metastasis. There were 1,987 (39.96%) patients with nutritional risk assessed by NRS2002 partial excluding weight loss information and 2,304 (46.34%) patients with nutritional risk assessed with NRS2002 including weight loss information (chi-squared test, *P* < 0.05). Four inflammatory indicators, NLR, ALI, PLR, and NRI, play a significant role in distinguishing patients with cachexia from those without cachexia (*P* < 0.05). Patients with cachexia showed higher NLR, higher PLR, lower NRI, and lower ALI, which was consistent with the observations in previous studies (22–25).

**Table 1:** Baseline characteristics of the study population.

**Characteristic**	**Overall (*n* = 4,712)**	**No-cachexia (*n* = 3,320)**	**Cachexia (*n* = 1,392)**	***P*-value**
Sex: male (*n*, %)	3,139 (66.6)	2,228 (67.1)	911 (54.4)	0.279
Comorbidities: yes (*n*, %)	1,844 (39.1)	1,268 (38.2)	576 (41.4)	0.043
Family cancer history: yes (*n*, %)	744 (15.8)	543 (16.3)	201 (14.4)	0.105
Smoking: yes (*n*, %)	2,820 (59.8)	1,971 (59.4)	849 (61.0)	0.313
**Tumor stage (** ***n*** **, %)**				< 0.001
I	368 (7.8)	291 (8.8)	77 (5.5)	
II	732 (15.5)	571 (17.2)	161 (11.6)	
III	1,268 (26.9)	918 (27.6)	350 (25.1)	
IV	2,344 (49.7)	1,540 (46.4)	804 (57.8)	
**Pathology (** ***n*** **, %)**				< 0.001
NSCLC	2,410 (51.1)	1,647 (49.6)	763 (54.8)	
SCLC	602 (12.8)	465 (14.0)	137 (9.8)	
Unknown	1,700 (36.1)	1,208 (36.4)	492 (35.3)	
**Feed (** ***n*** **, %)**				< 0.001
As usual	3,027 (64.2)	2,512 (75.7)	515 (37.0)	
Decreased	1,685 (35.8)	808 (24.3)	877 (63.0)	
Anorexia (*n*, %)	1,030 (21.8)	462 (13.9)	568 (40.8)	< 0.001
Vomiting (*n*, %)	236 (5.0)	90 (2.7)	146 (10.5)	< 0.001
Diarrhea (*n*, %)	98 (2.1)	57 (1.7)	41 (2.9)	0.01
Other symptoms (*n*, %)	1,513 (32.1)	830 (25.0)	683 (49.1)	< 0.001
**Activity (** ***n*** **, %)**				< 0.001
1	3,132 (66.5)	2,440 (73.5)	692 (49.7)	
2	1,204 (25.6)	732 (22.0)	472 (33.9)	
3	184 (3.9)	85 (2.5)	99 (7.1)	
4	161 (3.4)	57 (1.7)	104 (7.5)	
5	31 (0.6)	6 (0.2)	25 (1.8)	
Age (years), *M* (IQR)	61 [54, 67]	60 [53, 66]	62 [55, 68]	< 0.001
Height (cm), *M* (IQR)	165 [160, 170]	165 [160, 170]	165 [158, 170]	< 0.001
Weight (kg), *M* (IQR)	61 [55, 69]	63.9 [57.6, 70]	55 [49, 62]	< 0.001
BMI (kg/m^2^), *M* (IQR)	22.66 [20.6, 24.87]	23.45 [21.56, 25.51]	20.26 [18.36, 22.6]	< 0.001
KPS, *M* (IQR)	90 [80, 90]	90 [80, 90]	90 [80, 90]	< 0.001
Creatinine (μmol/L), *M* (IQR)	67.95 [58, 79.5]	28.1 [58.78, 79.6]	66.8 [56, 79.05]	0.293
Total protein (g/L), *M* (IQR)	68.9 [64.3, 73.3]	69.2 [64.8, 73.5]	68 [63.28, 72.7]	< 0.001
Albumin (g/L), *M* (IQR)	39.4 [36, 42.3]	40 [36.9, 42.83]	37.6 [33.9, 41.1]	< 0.001
Prealbumin (mg/L), *M* (IQR)	220 [180, 259.05]	227 [190, 264]	203.8 [156.08, 236.48]	< 0.001
BuN (mmol/L), *M* (IQR)	5.14 [4.17, 6.3]	5.19 [4.23, 6.31]	5.04 [4.00, 6.23]	0.951
TBil (μmol/L), *M* (IQR)	10.2 [7.6, 13.4]	10.3 [7.7, 13.5]	10 [7.3, 13.4]	0.595
DBil (μmol/L), *M* (IQR)	2.8 [2.04, 3.9]	2.8 [2, 3.8]	3 [2.1, 4.3]	< 0.001
AST, *M* (IQR)	22 [17.6, 28]	22 [18, 28]	21.8 [17, 29]	0.065
ALT (U/L), *M* (IQR)	19.8 [13.7, 30]	20 [14, 30]	19 [12.58, 30]	0.396
Blood glucose (mmol/L), *M* (IQR)	5.33 [4.88, 6.02]	5.35 [4.9, 6.01]	5.27 [4.8, 6.03]	0.324
Hemoglobin (g/L), *M* (IQR)	129 [116, 141]	132 [120, 143]	123 [110, 136]	< 0.001
RBC (^*^10^12^/L), *M* (IQR)	4.31 [3.88, 4.7]	4.38 [3.97, 4.75]	4.14 [3.69, 4.56]	< 0.001
WBC (^*^10^9^/L), *M* (IQR)	6.37 [5, 8.22]	6.23 [4.95, 7.95]	6.8 [5.16, 8.87]	< 0.001
Neutrophil (^*^10^9^/L), *M* (IQR)	4.14 [2.89, 5.83]	3.96 [2.82, 5.5]	4.62 [3.19, 6.7]	< 0.001
Lymphocyte (^*^10^9^/L), *M* (IQR)	1.5 [1.10, 1.93]	1.54 [1.14, 1.99]	1.39 [0.97, 1.8]	0.211
PLT (^*^10^9^/L), *M* (IQR)	231 [180, 292]	227 [177.75, 283]	247 [188, 310]	< 0.001
MAC (cm), *M* (IQR)	26.14 [24.4, 28.5]	27 [25, 29]	25 [23, 27]	< 0.001
TSF (mm), *M* (IQR)	15 [10, 20]	15.1 [11, 20.5]	13.7 [8, 18]	< 0.001
HGS (kg), *M* (IQR)	24.14 [19.7, 31.3]	25.2 [20.3, 32.5]	22.1 [17.78, 27.8]	< 0.001
MAMC (cm), *M* (IQR)	21.86 [20.08, 24.09]	22.22 [20.36, 24.31]	21.12 [19.12, 23.29]	< 0.001
CC (cm), *M* (IQR)	33 [31, 35.5]	34 [31.68, 36]	31.5 [29, 33.5]	< 0.001
EORTC QLQ-C30, *M* (IQR)	48 [43, 55.25]	47 [43, 53]	52 [46, 61]	< 0.001
NRS2002-partial, *M* (IQR)	2 [1, 4]	1 [1, 3]	4 [2, 4]	< 0.001
NLR, *M* (IQR)	2.72 [1.79, 4.38]	2.53 [1.69, 3.97]	3.33 [2.14, 5.61]	< 0.001
PLR, *M* (IQR)	154.68 [109.59, 223.48]	147.09 [106.27, 209.65]	179.83 [124.66, 261.77]	< 0.001
NRI, *M* (IQR)	104.42 [97.09, 111, 13]	106.79 [100.21, 113.17]	97.72 [90.01, 104.60]	< 0.001
ALI, *M* (IQR)	327.78 [188.76, 528.31]	373.82 [226.42, 578.16]	229.39 [124.77, 379.61]	0.002

ALI, advanced lung cancer inflammation index, the ALI was calculated from the following formula: BMI^*^Albumin/NLR; BMI, body mass index; ALT, alanine aminotransferase; AST, aspartate aminotransferase; BMI, body mass index; BuN, blood urea nitrogen; CC, calf circumference; DBil, direct bilirubin; EORTC, European Organization for Research and Treatment of Cancer; EORTC QLQ-C30, The EORTC QLG Core Questionnaire; HGS, hand grip strength; KPS, Karnofsky Performance Score; MAC, mid-arm circumference; MAMC, mid-arm muscle circumference; NLR, neutrophil-to-lymphocyte ratio; NLR, neutrophil count/lymphocyte count; NRI, nutritional risk index; NRS-2002 partial, Nutritional Risk Screening 2002 partial; NRI, nutritional risk index, the NRI was calculated from the following formula: 1.519^*^Albumin + 41.7^*^present body weight/ideal body weight. The ideal body weight is defined as (height – 100) ^*^0.9; NSCLC, non-small cell lung cancer; PLR, platelet-to-lymphocyte ratio; PLT, platelet; RBC, red blood cell; SCLC, small cell lung cancer; TBil, total bilirubin; TSF, Triceps skinfold thickness; WBC, white blood cell.

The EORTC QLG Core Questionnaire (EORTC QLQ-C30) was a 30-item instrument meant to assess some of the different aspects that define the quality of life of cancer patients.

### 3.2 Feature selection using the least absolute shrinkage and selection operator (LASSO)

**Figure 2:**
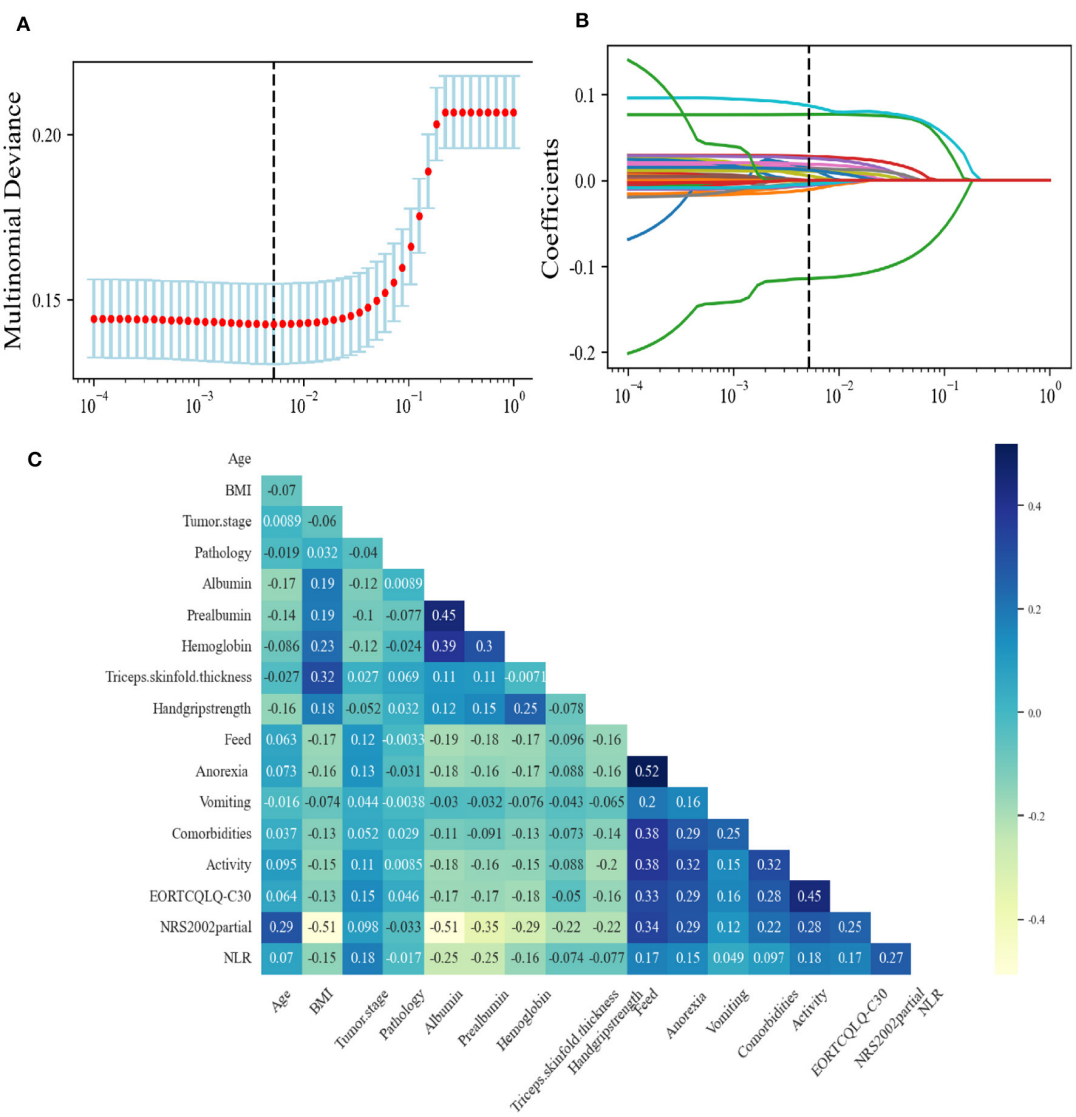
Feature selection using the Least Absolute Shrinkage and Selection Operator (LASSO). **(A)** The LASSO coefficient profiles and cross-validation for the classification model. **(B)** The Lasso regression coefficient path diagram. **(C)** Pearson's correlation analysis of the 17 variables. BMI, body mass index; EORTC, European Organization for Research and Treatment of Cancer; EORTC QLQ-C30, The EORTC QLG Core Questionnaire; NLR, neutrophil-to-lymphocyte ratio; NRS-2002 partial, nutritional Risk Screening 2002 partial.

The study population was divided into a training set (*n* = 3,298, 70%) for model derivation and a test set (*n* = 1,414, 30%) for assessing model performance. The LASSO (26) approach with 10-fold cross-validation was used to eliminate redundant features before modeling (Figures 2A, B). LASSO can shrink the coefficients of some variables by introducing a penalty term. It is an excellent method for processing high-throughput data and can effectively filter variables. The GBC model is an ensemble learning model based on decision tree and has strong generalization ability. The optimal model, based on 17 variables selected from the training and test sets, is detailed in Table 2. The 17 features selected by LASSO were BMI, feed, NRS2002 partial, NLR, EORTC QLQ_C30, prealbumin, handgrip strength, albumin, anorexia, hemoglobin, vomiting, activity, pathology, TSF, tumor stage, age, and comorbidity. Pearson's correlation analysis indicated no collinearity/multicollinearity among the LASSO-selected features (Figure 2C), supporting their use in subsequent model establishment.

**Table 2:** Study features used for modeling.

**Characteristic**	**Overall (*n* = 4,712)**	**Training (*n* = 3,298)**	**Test (*n* = 1,414)**	***P*-value**
Cachexia: yes (*N*, %)	1,392 (30.5)	1,006 (30.5)	386 (27.3)	0.03
**Tumor stage (N, %)**				0.942
I	368 (7.8)	253 (7.7)	115 (8.1)	
II	732 (15.5)	510 (15.5)	222 (15.7)	
III	1,268 (26.9)	888 (26.9)	380 (26.9)	
IV	2,344 (49.7)	1,647 (49.9)	697 (49.3)	
**Pathology (** ***N*** **, %)**				0.884
NSCLC	2,410 (51.1)	1,679 (50.9)	731 (51.7)	
SCLC	602 (12.8)	423 (12.8)	602 (42.6)	
Unknown	1,700 (36.1)	1,196 (36.3)	504 (35.6)	
**Feed (** ***N*** **, %)**				0.348
As usual	3,027 (64.2)	2,104 (63.8)	923 (65.3)	
Decreased	1,685 (35.7)	1,194 (36.2)	491 (34.7)	
Anorexia (*N*, %)	1,030 (21.9)	731 (22.2)	299 (21.1)	0.461
Vomiting (*N*, %)	236 (5.0)	179 (5.4)	57 (4.0)	0.052
Other symptoms (*N*, %)	1,513 (32.1)	1,076 (32.6)	437 (30.9)	0.26
**Activity (** ***N*** **, %)**				0.275
1	3,132 (66.5)	2,196 (66.6)	936 (66.2)	
2	1,204 (25.6)	823 (25.0)	381 (27.0)	
3	184 (3.9)	134 (4.0)	50 (3.5)	
4	161 (3.4)	121 (3.7)	40 (2.8)	
5	31 (0.6)	24 (0.7)	7 (0.5)	
Age (years), *M* (IQR)	61 [54, 67]	60 [53, 67]	61 [54, 67]	0.37
BMI (kg/m^2^), *M* (IQR)	22.66 [20.60, 24.87]	22.6 [20.52, 24.8]	22.83 [20.76, 25]	0.044
Albumin (g/L), *M* (IQR)	39.4 [36, 42.3]	39.39 [36, 42.3]	39.5 [36.07, 42.4]	0.964
Prealbumin (mg/L), *M* (IQR)	220 [180, 259.05]	220 [180, 257.85]	220 [180, 260]	0.717
Hemoglobin (g/L), *M* (IQR)	129 [116, 141]	130 [116, 141]	129 [117, 141]	0.613
TSF (mm), *M* (IQR)	15 [10, 20]	15 [10, 20]	15 [10, 20]	0.908
HGS (kg), *M* (IQR)	24.14 [19.7, 31.3]	24.14 [19.5, 31.2]	24.14 [20.1, 31.5]	0.49
EORTC QLQ-C30, *M* (IQR)	48 [43, 55.25]	48 [44, 55]	49 [43, 56]	0.777
NLR, *M* (IQR)	2.72 [1.79, 4.38]	2.71 [1.79, 4.40]	2.73 [1.79, 4.34]	0.84
NRS2002_partial, *M* (IQR)	2 [1, 4]	2 [1, 4]	2 [1, 4]	0.198

HGS, hand grip strength; KPS, Karnofsky Performance Score; NRI, nutritional risk index. Activity: 1: patients' activity was as usual; 2: patients' activity was slightly limited; 3: patients do not want to get up and move most of the time but do not spend more than half a day in bed or in a chair; 4: patients have little or no activity and spend more than half a day in bed or in a chair; 5: the patient was bedridden and unable to get up. Other symptoms refer to symptoms of the digestive tract other than anorexia and vomiting, including dry mouth, dysphagia, diarrhea, and constipation.

**Figure 3:**
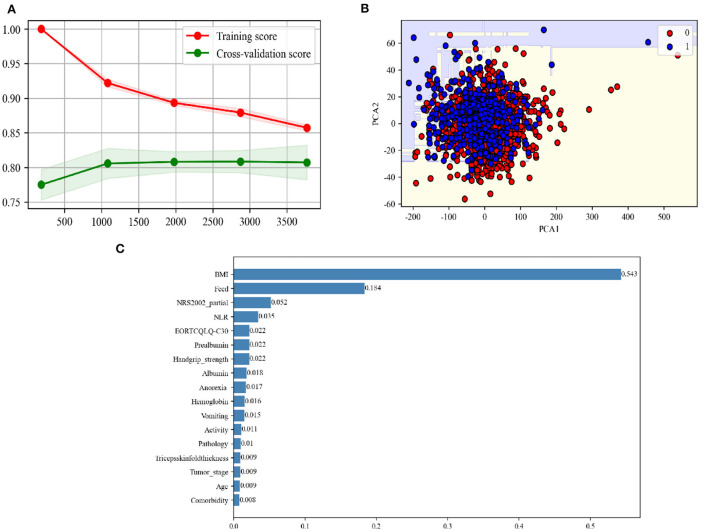
**(A)** Learning curve of the GBC using the training data. **(B)** Decision boundary of the GBC using the test data. PCA, principal component analysis. **(C)** The feature importance in the GBC ensemble learning model using the test data.

The learning curve showed that, when the amount of data increased, the GBC model had a tendency to converge, with the score converging at ~0.85 (Figure 3A). The decision boundary of the GBC model in the validation set (Figure 3B) showed the non-linear boundary in GBC model classification of cachexia and non-cachexia. The feature importance diagram (Figure 3C) showed the contribution of each feature to the model. Feature importance analysis showed that BMI was the most important feature in the GBC ensemble learning model (Figure 3C). The top five features that contribute most to the GBC model include feed, NRS2002 partial, NLR, and EORTCQLQ-C30. At present, the standardized nutritional support therapy steps recommended by domestic and foreign guidelines include nutritional screening, nutritional assessment, and nutritional intervention and monitoring (27). Nutritional screening is the first step. The “nutritional risk” derived from NRS2002 relevant to patient clinical outcomes displayed a basis in evidence-based medicine and had been validated in retrospective and prospective clinical studies and was currently the preferred screening tool recommended by many guidelines (28–30). QLQ-C30 consists of 30 questions to measure the quality of life of patients from function and symptoms and was the most widely used international method for measuring the quality of life in cancer patients (31). In addition, low BMI, reduced food intake, which was recognized in the clinical practice, and a marker of systemic inflammation that had been shown in the studies to be significantly related to nutrition and prognostic NLR (32, 33) contributed significantly to the GBC model for predicting cachexia.

### 3.3 The performance of different machine learning models and performance demonstration of the GBC model

We independently developed 18 types of machine learning models to predict the response labels using the training data (Table 3). The training set underwent 10-fold cross-validation, and the effectiveness of 18 models was compared by seven verification criteria, namely, accuracy, AUC, precision, recall, F1-score, MCC, and kappa. Gradient boosting classifier (GBC) and CatBoost classifier exhibited the highest AUC (0.854 vs. 0.853). The F1 score was used to further compare the performances of the machine learning models. The F1-score was used to measure the accuracy of unbalanced data sets, and it is the harmonic mean of precision and recall (34, 35). The F1-score of the GBC model was higher than that of the CatBoost classifier (0.658 vs. 0.654). In addition to the F1-score, the accuracy, precision, MCC, and kappa consistently indicate that the GBC model was optimal (Table 3, Supplementary Figure S1). We then evaluated the performance of the top five models, with the highest efficiency observed in the training set compared to the test set, as shown in Table 4. We can see that the GBC model also performed well in the test set (AUC = 0.859, accuracy = 0.818, precision = 0.801, recall = 0.550, F1 score = 0.652, MCC = 0.552, and kappa = 0.535). We thus selected the GBC model for future use.

**Table 3:** Metrics of performance for different ensemble learning models.

**Model**	**Accuracy**	**AUC**	**Precision**	**Recall**	**F1-score**	**MCC**	**Kappa**
Gradient boosting classifier	0.819	0.854	0.771	0.574	0.658	0.549	0.538
CatBoost classifier	0.815	0.853	0.76	0.576	0.654	0.542	0.531
Extra trees classifier	0.812	0.849	0.733	0.606	0.661	0.539	0.533
Random forest classifier	0.813	0.848	0.76	0.561	0.644	0.533	0.521
Linear discriminant analysis	0.82	0.847	0.739	0.639	0.684	0.563	0.559
Ridge classifier	0.82	0.846	0.747	0.62	0.677	0.559	0.554
Supportive vector machine—linear kernel	0.822	0.845	0.754	0.613	0.676	0.56	0.554
Light gradient boosting machine	0.809	0.845	0.738	0.584	0.651	0.53	0.522
Adaboost classifier	0.814	0.844	0.754	0.576	0.652	0.537	0.528
Multiple layer perceptron classifier	0.797	0.838	0.722	0.6	0.638	0.518	0.502
Logistic regression	0.815	0.836	0.736	0.612	0.667	0.545	0.54
Extreme gradient boosting	0.804	0.836	0.724	0.576	0.64	0.515	0.507
Quadratic discriminant analysis	0.783	0.82	0.678	0.548	0.604	0.464	0.458
Gaussian NB	0.777	0.815	0.644	0.599	0.619	0.463	0.462
Decision tree classifier	0.747	0.704	0.582	0.595	0.588	0.406	0.405
Bernoulli NB	0.723	0.691	0.59	0.295	0.392	0.262	0.238
Supportive vector machine—Radial Kernel	0.697	0.676	0.592	0.051	0.092	0.096	0.043
K neighbors classifier	0.704	0.657	0.522	0.331	0.405	0.231	0.221
Dummy classifier	0.695	0.5	0	0	0	0	0

ROC, receiver operating curve; AUC, area under the curve; MCC, Matthews correlation coefficient; NB, Naïve Bayes.

**Table 4:** Metrics of performance for different ensemble learning models in the test set.

**Model**	**Accuracy**	**AUC**	**Precision**	**Recall**	**F1-score**	**MCC**	**Kappa**
Gradient boosting classifier	0.818	0.859	0.801	0.55	0.652	0.552	0.535
CatBoost classifier	0.818	0.856	0.794	0.555	0.653	0.55	0.535
Extreme gradient boosting	0.821	0.859	0.782	0.589	0.672	0.563	0.553
Linear discriminant analysis	0.82	0.857	0.81	0.546	0.652	0.556	0.537
Random forest classifier	0.818	0.857	0.762	0.598	0.67	0.554	0.546

ROC, receiver operating curve; AUC, area under the curve; MCC, Matthews correlation coefficient.

**Figure 4:**
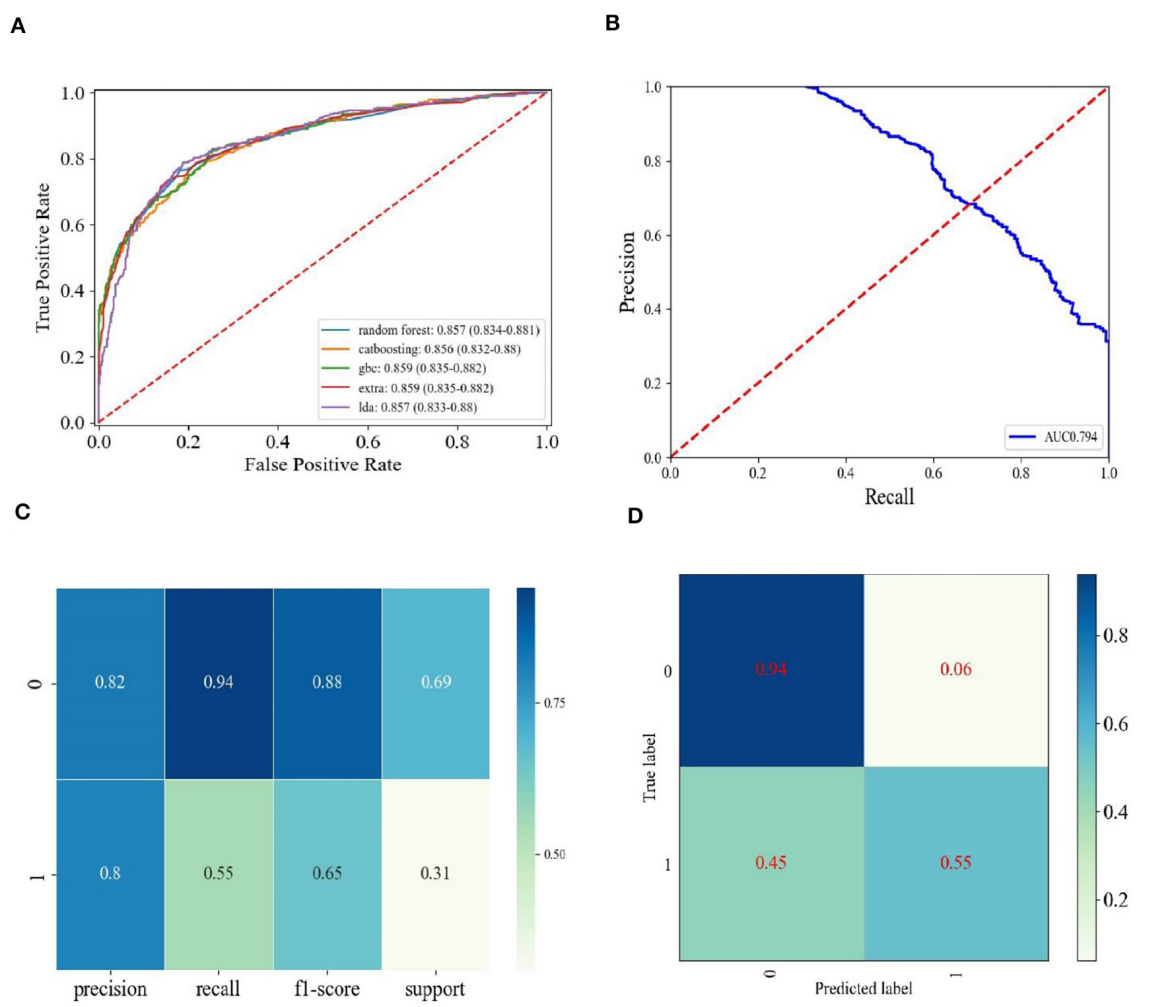
The performance demonstration of the GBC model. **(A)** ROC curves of the top five models with the best performance in the training set in the test set. Random forest, Random Forest Classifier; catboosting, CatBoost Classifier; GBC, Gradient Boosting Classifier; extra, Extreme Gradient Boosting; lda, Linear Discriminant Analysis. **(B)** Precision-recall curve for the GBC model in the test data. **(C)** Classification report for the GBC model using the test data. **(D)** Confusion matrix for the GBC using the test data.

The receiver operating characteristic (ROC) curve showed that the AUC was 0.859 (Figure 4A). The area under Precision Recall (PR) curves was 0.794 (Figure 4B). The GBC model classification report of the test set is shown in Figure 4C. The confusion matrix showed that, among 91% patients (no cachexia), 55% patients (cachexia) were correctly predicted (Figure 4D). Among all the indicators, the GBC model showed significant advantages in predicting cachexia in lung cancer patients who could not provide weight loss information, and it was expected to provide clinical diagnosis of cachexia in these patients.

## 4 Discussion

This study was a retrospective cohort investigation of 4,712 lung cancer patients at multi-centers in China. The study focused on a real-world clinical challenge, which predicted the onset of cancer cachexia in advance before significant weight loss. To our knowledge, this study represents the initial large-scale investigation tackling this challenge through ensemble learning methods grounded in traditional clinical characteristics. Our discoveries offer valuable insights that may assist clinicians or nutritionists in decision-making for the treatment of high-risk lung cancer patients, guiding effective management strategies to enhance patients' outcomes.

Weight loss was a major factor in the diagnosis of cachexia (12, 36). However, due to the lack of self-awareness in patients' daily life, the delay in recalling weight loss information and the inaccuracy of recalling made the diagnosis of cachexia difficult (37). Previous evidence showed that, once patients lose more than 5% of their body weight, the mortality rate had increased significantly (9). In addition, notable instances of treatment-related toxicity and increased mortality were observed in obese patients with low muscle mass, a condition referred to as sarcopenia obesity (38, 39). Clinical identification of these patients posed a challenge, especially since sarcopenia can be concealed by obesity body mass index (BMI) (40). Consequently, beyond weight loss, increased need for sensitive criteria arises to detect patients in the early stages of cachexia. Such assessments necessitate measurements beyond standard body weight, including instruments for evaluating muscle mass and/or physical activity (38). In the context of cancer, because of the complexity of body composition, some researchers suggested that measurements of specific body compartments using methods such as computed tomography should outweigh the role of weight loss in evaluating cachexia test results (41). We used a convenient and cost-effective GBC ensemble learning model that incorporated demographic, anthropometric, and laboratory parameters, excluding baseline weight loss to construct an effective predictive model for early identification of cachexia. A total of 18 machine learning models were evaluated, and a GBC ensemble learning model with optimal performance was obtained. Compared to the training set, the GBC model showed good performance in the testing set.

In our study, we eliminated weight loss, a dynamically changing feature, and only used currently measurable characteristics to construct a diagnostic model of cachexia. Low BMI, reduced food intake, digestive symptoms, and NLR contributed more to the GBC model. Currently, the GLIM framework suggests the inclusion of at least one phenotypic criterion and one etiological criterion for diagnosing malnutrition. Etiological criteria encompass reduced food intake, digestive or absorption disorders, inflammation, or disease burden in patients (42). BMI was the most important indicator to measure the nutritional status of the human body and is designated to be one of the diagnostic criteria for cachexia (12). The mechanism of anorexia involved tumor-derived humoral factors that can induce cancer anorexia by modulating neuropeptide hormones in the brain associated with eating (43). Furthermore, the elucidation of the process indicates that anorexia preceded tissue wasting in cachexia. In a previous study, severe and moderate reduction in food intake was also found to independently predict OS. When anorexia, vomiting, or other digestive symptoms occurred or food intake decreased, patients were at higher risk for cachexia, which was consistent with findings of previous research (44). In the tumor context, systemic inflammation played an important role in the onset and progression of cancer, existed during cancer-associated cachexia, and served as a diagnostic tool for cancer-associated cachexia (25, 45). Recent meta-analyses demonstrated an association between elevated NLR and reduced progression-free survival and OS both in NSCLC and SCLC (33, 46). A prior study identified an association between elevated NLR and weight loss, as well as an increased prevalence of cachexia in cancer patients with advanced colon, lung, or prostate cancer (22). It had also been found that high levels of NLRs at baseline and a progressive increase in NLRs during treatment were associated with progressive disease, low OS, and weight loss in NSCLC patients (47). In our GBC model, NLR was one of the top five clinical features contributing to the model, indicating the important value of NLR in the diagnosis of lung cancer cachexia.

However, this study had several potential limitations. First, the weight loss relied on patient's reported historical weight, introducing the possibility of recalling bias. To mitigate this, only data from patients who were able to provide past weight information were included. Second, the ASMI in this study was derived from an anthropometric equation validated for the Chinese population. While the equation had demonstrated good agreement with dual-energy X-ray imaging and anthropometric data were readily available (17). It is essential to note that more precise muscle mass measurements for diagnosing cachexia may be obtained through imaging techniques such as dual-energy x-ray imaging or bioimpedance analysis (48). However, our GBC model showed that the classification results of the ASMI were of relatively little importance in the diagnosis of cachexia. Imaging indicators that could more accurately assess muscle mass might improve the performance of our model. For example, transverse CT images, typically at standard markers frequently presented in abdominal CT (such as the third lumbar spine), had established correlations with DXA equivalent total body fat and muscle mass (38, 49). Future studies using more advanced techniques to measure muscle mass are required. Third, as an etiological indicator of cachexia, inflammatory indicators are prognostic indicators of advanced lung cancer, as observed in previous studies. One potential serum biomarker commonly used in clinical practice was C-reactive protein (CRP) (25) that is used to identify patients at risk for cancer cachexia, when combined with other weight loss and nutrient intake factors (50). Due to the substantial amount of missing data on CRP, we could not include CRP and its related systemic inflammation indicators, which would be further improved in the subsequent data collection. However, in our study, we also demonstrated a significant correlation between NLR/ALI/PLR and cachexia through a univariate analysis (*P* < 0.05). Our model also included NLR, a marker of systemic inflammation, which could compensate for the missing CRP data to some extent. In future studies, we could analyze and clarify the role of systemic inflammatory indicators in the diagnosis of cachexia.

In conclusion, the GBC ensemble learning model using clinical data without weight loss information could identify patients with lung cancer cachexia. Feature importance showed the contribution value of each clinical feature to the diagnosis of cachexia. This study may support patient counseling and targeted interventions to perform nutritional treatment in advance and improve patient prognosis and life quality.

## Data availability statement

The raw data supporting the conclusions of this article will be made available by the authors, without undue reservation.

## Ethics statement

The studies involving humans were approved by the Institutional Review Committee of Beijing Shijitan Hospital. The studies were conducted in accordance with the local legislation and institutional requirements. The participants provided their written informed consent to participate in this study.

## Author contributions

PJ: Data curation, Funding acquisition, Project administration, Supervision, Validation, Writing—original draft, Writing—review & editing. QZ: Investigation, Methodology, Writing—review & editing, Writing—original draft. XWu: Data curation, Investigation, Methodology, Writing—original draft, Writing—review & editing. FS: Data curation, Methodology, Writing—original draft, Writing—review & editing. KS: Investigation, Methodology, Writing—original draft, Writing—review & editing. XWa: Data curation, Investigation, Methodology, Writing—original draft, Writing—review & editing.
