# Supplementary material for: Identification of cachexia in lung cancer patients with an ensemble learning approach
Source: Front Nutr. 2024 May 30;11:1380949. doi: 10.3389/fnut.2024.1380949 (PMC11169803; doi:10.3389/fnut.2024.1380949)
Supplement: Supplementary file 1 [file Image_1.pdf]

Figure S1

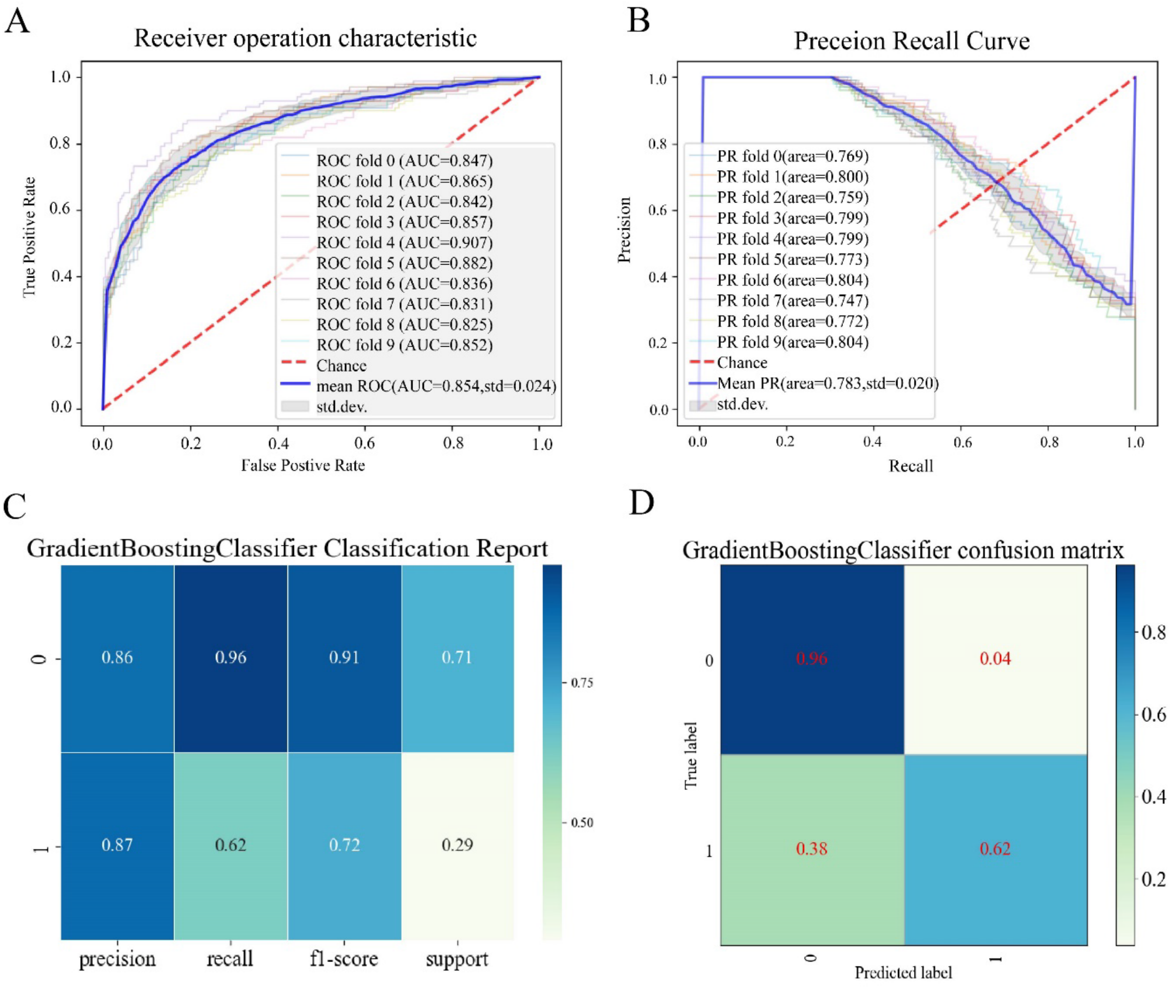

Figure S1. Metrics of performance for the gradient boosting classifier (GBC) in training set. (A) Receiver operation characteristic (ROC) curve for the GBC in the training data using10-fold cross-validation. (B) Precision-recall curve for the GBC in the training data using10-fold cross-validation. (C) Classification report for the GBC in the training data. (D) Confusion matrix for the GBC in the training data.
